# The association between plasma choline, growth and neurodevelopment among Malawian children aged 6–15 months enroled in an egg intervention trial

**DOI:** 10.1111/mcn.13471

**Published:** 2022-12-25

**Authors:** Megan G. Bragg, Elizabeth L. Prado, Bess L. Caswell, Charles D. Arnold, Matthews George, Lisa M. Oakes, Aaron G. Beckner, Michaela C. DeBolt, Brian J. Bennett, Kenneth M. Maleta, Christine P. Stewart

**Affiliations:** ^1^ Department of Nutrition University of California Davis Davis California USA; ^2^ USDA Western Human Nutrition Research Center Davis California USA; ^3^ School of Public Health and Family Medicine Kamuzu University of Health Sciences Blantyre Malawi; ^4^ Center for Mind and Brain University of California Davis Davis California USA; ^5^ AJ Drexel Autism Institute Drexel University Philadelphia Pennsylvania USA

**Keywords:** Africa, child development, choline, complementary feeding, eggs, growth, infant nutrition sciences

## Abstract

Choline is an essential micronutrient that may influence growth and development; however, few studies have examined postnatal choline status and children's growth and development in low‐ and middle‐income countries. The aim of this observational analysis was to examine associations of plasma choline with growth and development among Malawian children aged 6–15 months enrolled in an egg intervention trial. Plasma choline and related metabolites (betaine, dimethylglycine and trimethylamine N‐oxide) were measured at baseline and 6‐month follow‐up, along with anthropometric (length, weight, head circumference) and developmental assessments (the Malawi Developmental Assessment Tool [MDAT], the Infant Orienting with Attention task [IOWA], a visual paired comparison [VPC] task and an elicited imitation [EI] task). In cross‐sectional covariate‐adjusted models, each 1 SD higher plasma choline was associated with lower length‐for‐age z‐score (−0.09 SD [95% confidence interval, CI −0.17 to −0.01]), slower IOWA response time (8.84 ms [1.66–16.03]) and faster processing speed on the VPC task (−203.5 ms [−366.2 to −40.7]). In predictive models, baseline plasma choline was negatively associated with MDAT fine motor z‐score at 6‐month follow‐up (−0.13 SD [−0.22 to −0.04]). There were no other significant associations of plasma choline with child measures. Similarly, associations of choline metabolites with growth and development were null except higher trimethylamine N‐oxide was associated with slower information processing on the VPC task and higher memory scores on the EI task. In this cohort of children with low dietary choline intake, we conclude that there were no strong or consistent associations between plasma choline and growth and development.

## Key messages

- Few studies have examined the role of choline for child growth and neurodevelopment in low‐ and middle‐income countries, where intake of choline is likely low and suboptimal growth and development are common.
- Plasma choline was not associated with most measures of growth or neurodevelopment among this sample of Malawian children; three of the four significant associations were in the direction of poorer growth and development with higher plasma choline.
- Further research is needed to understand the link between postnatal choline status, growth and neurodevelopment in diverse settings. Improved biomarkers of choline status are required across all settings.

## Graphical abstract

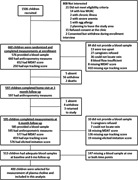

## 1 INTRODUCTION

Globally, more than 20% of children under 5 years were stunted in 2019, mostly in low‐ and middle‐income countries (LMICs; UNICEF WHO & World Bank, 2020), putting these children at risk for poor health outcomes and decreased adult productivity (Hoddinott et al., 2013). Additionally, nearly 250 million children younger than 5 years in LMICs are at risk for not reaching their developmental potential (Black et al., 2017). Nutrition is one factor which influences neurodevelopment and growth (Danaei et al., 2016; Prado & Dewey, 2014).

Choline is an essential micronutrient that may affect the risk of stunting and poor development (Bragg et al., 2021), perhaps through its role as a precursor for important metabolites including phosphatidylcholine, acetylcholine, trimethylamine N‐oxide (TMAO), betaine and dimethylglycine (DMG). Choline is crucial for brain development, especially for the development of the dentate gyrus region of the hippocampus (Albright et al., 1999; Blusztajn & Mellott, 2012; Zeisel, 2004), which is needed for declarative memory (Bauer et al., 2010). Rodent studies show consistent improvements in memory with perinatal supplementation of choline above standard feed levels (Blusztajn et al., 2017; McCann et al., 2006; Meck et al., 2008), including in models of developmental risk such as prenatal alcohol exposure (Thomas et al., 2010) and iron deficiency (Kennedy et al., 2014, 2018; Tran et al., 2016). In a human trial, choline supplementation above the Adequate Intake (AI) level during the third trimester of pregnancy was associated with improvements in children's processing speed and sustained attention through 7 years of age (Bahnfleth et al., 2022; Caudill et al., 2018).

Few studies have reported on the relationship between postnatal (<2 years) choline concentration and child growth and neurodevelopment in LMICs (Bragg et al., 2021). Because choline is mainly found in animal source foods, which are relatively expensive (Headey & Alderman, 2019), intake is likely suboptimal in many LMICs (Wiedeman, Barr, et al., 2018). The complementary feeding period may be particularly important to ensure adequate choline intake, as it is a period of rapid growth and hippocampal development (Seress & Abraham, 2008; Stewart et al., 2013). For young children, breast milk is a rich source of choline, although concentrations vary with maternal diet and genotype (Fischer et al., 2010). As children in LMICs transition from breast milk to traditional complementary foods, they may be at risk for low choline intake. On the other hand, common genetic polymorphisms, single‐nucleotide polymorphisms (SNPs), affect individual choline requirements (Ganz et al., 2017), and there is evidence for negative selection of SNPs that increase choline requirements in African populations with traditionally low choline intake (Silver et al., 2019).

The aim of this analysis was to examine the cross‐sectional and predictive associations between plasma choline and measures of child growth and neurodevelopment during the early complementary feeding period (6–15 months) in rural Malawi. Developmental assessments included measures of language, motor and personal–social milestones, which are indicators of global brain development and can detect general neurodevelopmental delay, as well as specific measures of memory and attention, two cognitive domains which have been specifically linked to choline in previous studies (Caudill et al., 2018; Cheatham & Sheppard, 2015). We hypothesised that plasma choline would be positively associated with anthropometric and developmental measures across this period. In exploratory analyses, we tested the effect of several potential effect modifiers, as well as the association between choline metabolites (plasma betaine, DMG and TMAO) and child growth and development.

This is a secondary analysis of data from a randomised trial in Malawi (the Mazira Project) which provided one egg per day for 6 months to children aged 6–9 months at enrolment. Eggs are a rich source of choline, and improvements in choline status were expected to mediate improvements in growth and development, similarly to an egg intervention trial in Ecuador (Iannotti, Lutter, Stewart, et al., 2017; Iannotti, Lutter, Waters, et al., 2017). However, there was no effect of eggs on most growth or developmental outcomes (Prado et al., 2020; Stewart et al., 2019), and no improvement in plasma choline concentration (Bragg et al., 2022) in Malawi. This analysis therefore serves a second purpose: by investigating the association between choline status and growth and development in this context, we may also gain a deeper understanding of the null results of the Mazira Project randomised trial.

## 2 METHODS

The Mazira Project randomised trial (clinicaltrials.gov: NCT03385252) took place in rural Malawi from February 2018 to January 2019. This trial investigated the effect of providing one egg per day versus a nonintervention control among 660 Malawian children. Children aged 6–9 months were individually randomised to intervention or control for 6 months. The intervention group received weekly batches of eggs, and caregivers were asked to feed the child one egg per day in addition to normal feeding. The control group received no eggs, and caregivers were asked to feed the child as they normally would. Both groups received twice‐weekly home visits, as well as information about food hygiene and handwashing. Descriptions of baseline characteristics by group are reported elsewhere (Stewart et al., 2019). Briefly, baseline egg consumption was low and similar between groups (4.0% in the control group and 4.2% in the egg group).

### 2.1 Participants

Children residing in the catchment areas of two health centres (Lungwena Health Center and St. Martins Rural Hospital in Malindi) were eligible to enrol. These areas are rural, with most families engaged in fishing and agricultural labour. Staff recruited age‐eligible children and caregivers during household visits. Exclusion criteria were: egg allergy, history of serious allergic reactions, congenital defects or conditions which may affect growth and development, severe anaemia (haemoglobin <5 g/dl), low mid‐upper arm circumference (<12.5 cm), presence of bipedal oedema or acute illness or injury warranting hospital referral. Children of families who planned to leave the study area within the next 6 months were also excluded.

### 2.2 Data collection

Detailed descriptions of data collection for this trial have been previously published (Prado et al., 2020; Stewart et al., 2019). Briefly, children and caregivers came to the study site at enrolment and at study end 6 months later. At both times, staff collected anthropometric, dietary, demographic and developmental data. Blood samples were collected to assess exclusion criteria, including tests for haemoglobin concentration (Hemocue 201, HemoCue Inc.) and malaria antigen (DF Bioline Malaria Ag P.f/Pan, Abbott Diagnostics). At the 6‐month follow‐up, the Family Care Indicators (FCI) interview was administered, which assesses children's opportunities for stimulation (Hamadani et al., 2010).

Other data were collected during home visits. Soon after enrolment, staff administered the Household Food Insecurity Access Scale (Coates et al., 2007) and Home Observation Measurement of the Environment (HOME) Inventory (Caldwell & Bradley, 2003) questionnaires and collected data on housing materials and animal ownership for incorporation into a housing and asset index. After 3 months of enrolment, study staff collected anthropometric and dietary data during a home visit. Throughout the study, caregivers reported weekly on child morbidity symptoms, including the number of days with diarrhoea. The longitudinal prevalence of diarrhoea was calculated as the number of days with reported diarrhoea divided by the total number of days of recall.

### 2.3 Anthropometric measures

Trained and standardised pairs of anthropometrists measured children's recumbent length (in cm) using a Holtain length board, weight (in kg) using a Seca 874 digital scale, and head circumference (in cm) using insertion tapes (Health Books International at enrolment and Seca model 212 at 6‐month follow‐up). World Health Organization Growth Standards were used to convert values to z‐scores (length‐for‐age [LAZ], weight‐for‐age [WAZ], weight‐for‐length [WLZ] and head circumference‐for‐age [HCAZ]) (WHO Multicentre Growth Reference Study Group, 2006).

In addition to continuous z‐scores, conditional and dichotomous measures were calculated, in line with best practices for linear growth analyses (Wit et al., 2017). To calculate conditional variables, anthropometric data collected at 6‐month follow‐up was regressed on data from enrolment and 3‐month follow‐up (Stein et al., 2010). The residuals reflect how each child's growth over the 6‐month study period differed from expected based on their initial growth status compared to the other study participants. A positive value reflects comparatively faster growth (or slower faltering); a negative value reflects comparatively slower growth (or quicker faltering). Because the insertion tapes were changed during the study, a conditional measure of head circumference was not included. Dichotomous outcomes were defined as being above or below a cutoff: stunted (LAZ ≤ −2), underweight (WAZ ≤ −2), wasted (WLZ ≤ −2) or small head circumference (HCAZ ≤ −2).

### 2.4 Developmental assessments

Four developmental assessments (two behavioural measures and two measures based on eye‐tracking) were conducted by trained and standardised data collectors.

#### 2.4.1 Behavioural measures

The Malawi Developmental Assessment Tool (MDAT) includes 136 items across four domains (fine motor, gross motor, personal social and language development) which are scored as pass/fail based on the child's performance (or, for the personal social domain, parental report). For each domain, z‐scores were calculated based on published Malawian norms (Gladstone et al., 2010). The MDAT has been validated for use in Malawi and has high sensitivity (97%) and specificity (82%) to detect neurodevelopmental impairment in this context (Gladstone et al., 2010).

In the elicited imitation task, children demonstrate declarative memory by imitating sequences of actions demonstrated by outcome assessors (Bauer, 2010). In our adaptation of the task, children were asked to imitate eight two‐action sequences (16 target actions, 8 ordered sequences) performed using sets of toys. For each set, assessors first recorded which actions the child performed before the demonstration (spontaneous actions) during a 30‐s free play. Then, the assessor performed the sequence twice while the child watched. Afterwards, the assessor scored the child's ability to reproduce the target actions (actions recalled score: 0–16) and sequences (sequences recalled score: 0–8) during two 30‐s imitation sessions. Information on the adaptation and piloting of this task has been published (Prado et al., 2020). In a few cases, children were unable to complete items due to fussiness, sleepiness, or missing or damaged toys; to correct for this, scores were calculated by multiplying the percent of correct actions or sequences available to the child by the maximum possible score. Children who were offered fewer than 8 actions or 4 sequences were excluded. The elicited imitation task was measured at 6‐month follow‐up only.

#### 2.4.2 Eye‐tracking measures

Children were seated on their caregiver's lap facing a monitor with an eye‐tracking system (Tobii Pro X2‐60) attached. The eye tracker recorded the *x* and *y* coordinates of the child's gaze on the screen 60 times per second by calculating the position of the pupil and corneal reflection. Two eye‐tracking stations were used at the study site, each including a laptop, monitor, webcam and eye tracker surrounded by 4 black curtains. At baseline, children who enroled before 4 April 2018 (39%) completed a pilot version of the eye‐tracking tasks, which was later revised; these children were not included in analysis of baseline eye‐tracking data.

The visual paired comparison (VPC) task measures children's recognition memory based on the concept of novelty preference, or children's preference to look at unfamiliar items. In this version of the test based on a previous study (Rose, 1983), children were presented with two identical stimuli (an African face) on the left and right sides of the screen for 20 s (the familiarisation period). Then, after a brief delay, the familiar face was shown on one side of the screen paired with a novel face on the other for 20 s, with the position of the faces reversed at 10 s (the recognition memory period). This was repeated with different faces four times. Two outcome measures were calculated from this data: a novelty preference score and the peak look length during familiarisation.

To create novelty preference scores, the number and length of fixations to each side of the screen were calculated using the Tobii I‐VT fixation filter. Fixations represent a period when infants' gaze position is stable and directed towards a specific focal point. For each trial, the novelty preference score is the percent of time spent fixating on the side of the screen containing the novel stimulus compared to the total time looking at the screen. Trials with <1 s of looking time during the familiarisation or recognition memory periods were excluded (11% of trials) since the child may not have been on‐task.

Peak look length was calculated as the duration of the longest look during the familiarisation phase of each trial. In previous studies, shorter looks were associated with improved attention and faster information processing (Frick et al., 1999); however, these studies used human scorers rather than eye‐tracking devices. To mimic the ability of human scorers to identify eye movements, fixations identified by the Tobii filter were recoded into ‘looks,’ which were defined as periods of visual attention towards one side of the screen that lasted ≥1 s and were not interrupted for longer than 1 s.

In the Infant Orienting with Attention (IOWA) task, children demonstrate their attentional processes by shifting their gaze towards targets appearing on the screen (Ross‐Sheehy et al., 2015). In this task, children were shown a central image (a smiley face), then a 100‐ms visual cue (a small black circle), followed by a 1000‐ms target (a picture of a colourful everyday object) on one side of the screen. Children's gaze was tracked as it shifted from the central image to the target, and the response time was defined as the time from the appearance of the target to the first fixation on that side of the screen. Trials with <200 ms of looking time at the central image were excluded (10% of trials), as the child was not properly fixated on the centre of the screen. Trials with response times <100 ms or >1000 ms were also excluded (1% of trials), as they may reflect eye movements that started before the appearance of the target or off‐track behaviour, respectively. The IOWA task included 96 trials across four conditions which varied by the location of the visual cue (same side as target, opposite side, both sides or not present).

### 2.5 Plasma metabolites

Venous blood was collected into lithium heparin tubes at baseline and 6‐month follow‐up. Samples were centrifuged within a mean of 28 (SD 42) min of collection. Plasma and cell samples were separated into aliquots, which were stored in the local freezer at −20°C within a mean of 37 (SD 14) min of centrifugation. Each afternoon, the aliquots were transported to the main laboratory for storage at −80°C.

Details of plasma choline measurement for this study have been described (Bragg et al., 2022). Briefly, plasma choline was measured at baseline and 6‐month follow‐up using two analysis methods. First, plasma choline was measured in a subsample of 400 children using ultra‐high performance liquid chromatography–tandem mass spectrometry (UPLC‐MS/MS) by Metabolon Inc. These semi‐quantitative data describe the distribution of plasma choline concentration and may be used for regression analysis; however, they are in relative intensity units. Betaine, DMG, TMAO and more than 800 other metabolites were also measured in this way. Additionally, plasma choline was measured quantitatively in a subsample of 60 children using liquid chromatography–tandem mass spectrometry (LC‐MS/MS) at the USDA Western Human Nutrition Research Center. These data provide the absolute concentration and can be used to compare to other studies; however, due to cost restraints, the sample size was limited, and these data were not used in regression analyses. Betaine and TMAO, but not DMG, were measured using similar validated and standardised protocols. Plasma concentrations using the two different methods were well correlated (choline: *r* = 0.92, betaine: *r* = 0.98, TMAO: *r* = 0.98; Bragg et al., 2022).

Other metabolites measured include plasma docosahexaenoic acid (DHA), leucine, C‐reactive protein (CRP), alpha(1)‐acid glycoprotein (AGP), ferritin and zinc. Plasma DHA and leucine were measured in relative intensity units using UPLC‐MS/MS by Metabolon Inc. CRP, AGP and ferritin were measured using enzyme‐linked immunoassay by the VitMin lab (Erhardt et al., 2004). Ferritin was adjusted for inflammation, as measured by CRP and AGP, using the Biomarkers Reflecting Inflammation and Nutritional Determinants of Anaemia (BRINDA) approach (Namaste et al., 2017). Plasma zinc was measured using inductively coupled plasma mass spectrometry at Washington University in St Louis.

### 2.6 Sample size

The sample size for the randomised trial was 660 children, based on the hypothesised difference in LAZ between groups at the end of the intervention, which was the pre‐specified primary outcome of the trial. This analysis included a subset of 400 children (200 per group) who provided adequate blood samples at baseline and 6‐month follow‐up and were randomly selected for semi‐quantitative (UPLC‐MS/MS) lab analysis. This sample size is sufficient to detect correlations between plasma metabolites and growth and developmental outcomes as small as 0.14 with 80% power and a two‐sided α of 0.05.

### 2.7 Statistical analysis

Statistical analysis plans were developed and shared publicly before analysis (https://osf.io/vfrg7/). To examine cross‐sectional associations between plasma choline and continuous growth and developmental outcomes, we pooled data from both time points and used plasma choline at the corresponding time point as a predictor in linear regression models, with time point as a covariate. Similarly, dichotomous outcomes were examined using logistic models with both time points. We used robust standard errors with participant as the independent unit to account for repeated measures within participant over time and multiple trials per participant for certain developmental outcomes (VPC novelty preference and peak look length, IOWA response time). To examine predictive associations of baseline plasma choline with growth and developmental measures at 6‐month follow‐up, we ran linear regression models with baseline plasma choline as a predictor. Each model was assessed for linearity, outliers and normality and homoscedasticity of residuals. Children with missing plasma, growth or developmental data were not included. All analyses are reported as the difference in growth or developmental outcome per 1 SD higher plasma choline, except logistic models, for which odds ratios are reported.

Minimally adjusted models included variables related to data collection, specifically: time of last food intake other than breast milk, water, or tea before blood draw, calendar month of blood draw and anthropometrist or developmental data assessor. Because eggs are rich in choline, group assignment was included; however, previous analyses show that plasma choline values were similar between groups at baseline and were not statistically significantly different between groups at the 6‐month follow‐up (Bragg et al., 2022). The time point of data collection was included in models that contained both time points. For developmental assessments, the child's mood, activity level and interaction with the assessor during tasks were included. For the elicited imitation actions recalled score, the ‘spontaneous actions’ score was included to account for actions performed independent of memory. For eye‐tracking tasks, an eye‐tracking station indicator was included. For the novelty preference score, the total time spent fixating on the screen during the familiarisation period was included. For IOWA response time, condition was included.

Covariates for fully adjusted models were pre‐specified based on a theoretical causal model framework, with the following variables assessed for inclusion: child age, sex and birth order, baseline maternal age and education category, baseline household asset index and baseline food insecurity category. For growth outcomes, additional covariates included plasma leucine and zinc; plasma inflammatory markers (CRP and AGP); longitudinal prevalence of diarrhoea; and maternal height. For developmental outcomes, additional covariates included baseline plasma inflammation‐adjusted ferritin and child stimulation (as measured by HOME at baseline and/or FCI at 6‐month follow‐up). Plasma DHA was not included as a covariate, as a portion of choline's effect on neurodevelopment may be via synergy with DHA (Mun et al., 2019). Breastfeeding may affect plasma choline levels, neurodevelopment and growth; however, it was not included in our statistical models due to nearly universal prevalence of breastfeeding (>99%) in the study sample throughout the study period. Variables associated with the growth or developmental outcome with *p* < 0.1 were retained in the final multivariable models.

Additionally, we tested the association of choline metabolites betaine, DMG and TMAO with growth and development in minimally adjusted, exploratory models, using similar methods as described for plasma choline. Finally, we tested potential effect modifiers of the associations between choline, growth and development in exploratory minimally adjusted models.

All analyses used two‐sided tests with an alpha of 0.05. Given the large number of exploratory tests, significant *p* values should be interpreted cautiously.

## 3 RESULTS

### 3.1 Participant characteristics

**Figure 1:**
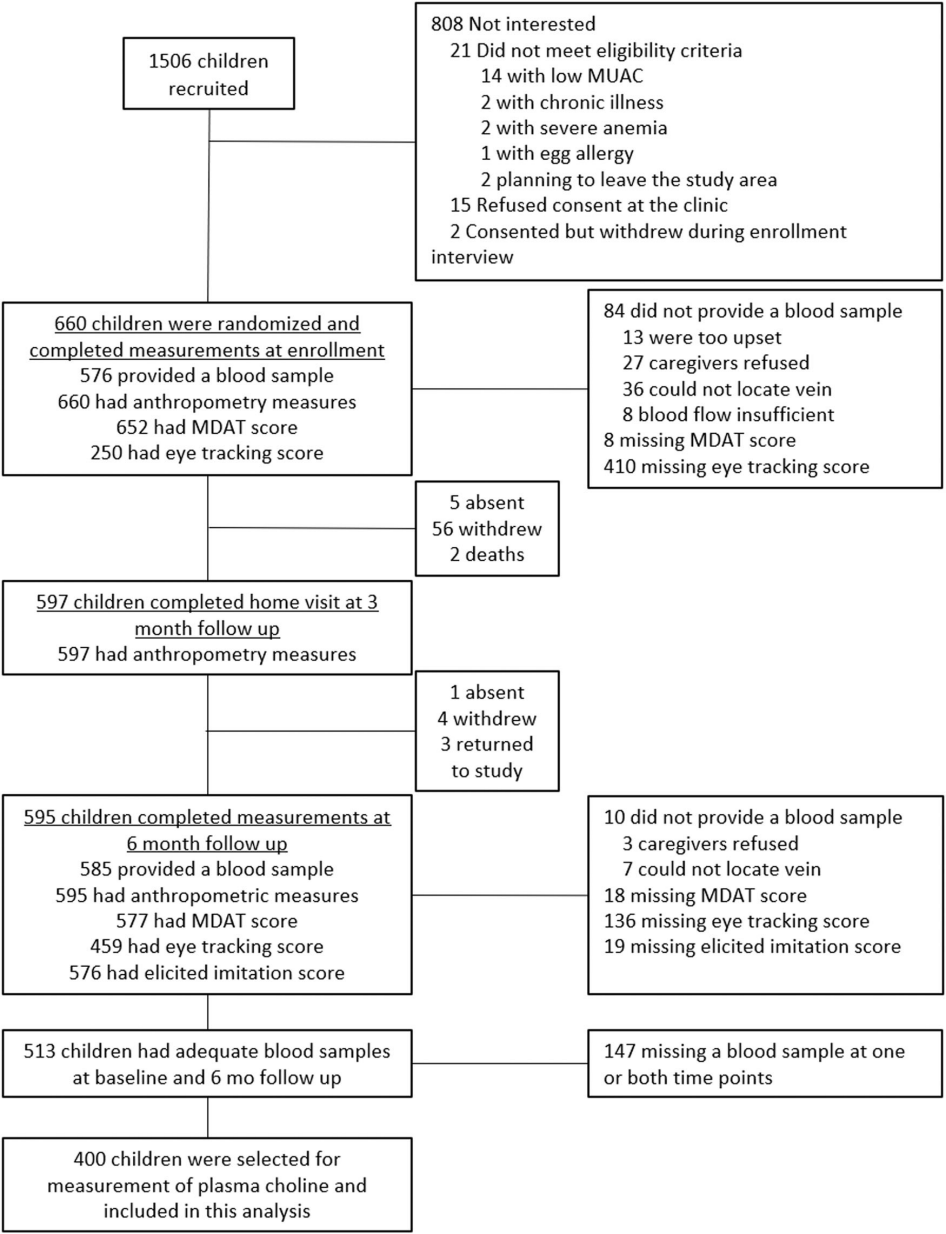
Study flow diagram for this secondary analysis of plasma choline, growth and development among young Malawian children

A total of 400 children were included in this analysis (Figure 1), distributed equally across the sexes (Table 1). Children in this sample were nearly universally breastfed. Although nearly a quarter of children consumed fish, as reported by caregivers on a 24‐h dietary recall at baseline, consumption of other animal source foods was <10%. Anaemia prevalence was high (62%). The mean longitudinal prevalence of diarrhoea was 10% (SD 10) of recall days. The majority of households reported moderate to severe food insecurity. Children in this analysis were similar to those who were in the main trial but excluded from this analysis (*n* = 260; Supporting Information: Table 1).

**Table 1:** Baseline characteristics of children enroled in the Mazira Project and included in this secondary analysis of plasma choline, development and growth (*n* = 400)

	Mean (SD) or (%)
*Child and maternal characteristics*	
Child age (mo)	7.4 (1.2)
Male (%)	53.3
First born (%)	27.8
Animal source food consumption (%)^a^	
Consumed dairy	9.8
Consumed meat	2.5
Consumed egg	3.8
Consumed fish	23.6
Any breast milk (%)	99.7
Anaemia prevalence (Hgb < 11 g/dl) (%)	62.2
Malaria prevalence (%)	14.5
Longitudinal prevalence of diarrhoea (proportion of days)	0.1 (0.1)
Maternal age (years)	26.0 (6.8)
Maternal BMI (kg/m^2^)	21.8 (3.0)
Maternal height (cm)	156.9 (5.2)
Mother completed primary school (%)	20.5
*Household characteristics*	
Number of household members	6.0 (2.6)
Moderate to severe food insecurity (%)^b^	78.0
Owns latrine (%)	96.5
Owns cows (%)	2.5
Owns goats (%)	20.0
Owns chickens (%)	33.3
HOME Inventory score	398
Family Care Indicators score^c^	400

Abbreviations: BMI, body mass index; Hgb, haemoglobin; HOME, Home Observation Measurement of the Environment.

^a^ As reported by caregiver on a 24‐h dietary recall.

^b^ As defined by the Household Food Insecurity Access Scale (Coates et al., 2007).

^c^ Measured at 6‐month follow‐up only.

Plasma choline decreased with age (*n* = 400, Supporting Information: Figure 1) and over the study period (*n* = 60, Table 2), from a mean (SD) of 17.1 (3.5) µmol/L at baseline to 14.6 (3.6) µmol/L at 6‐month follow‐up. Plasma betaine and TMAO increased with age and across the study period. Inflammation‐adjusted ferritin levels decreased from baseline to 6‐month follow‐up. DMG and leucine were not included in quantitative analyses, so absolute concentrations are unavailable.

**Table 2:** Plasma concentrations, developmental scores and growth measures for Mazira Project participants included in this secondary analysis

	Baseline (age 6–9 months)		3‐Month follow‐up (age 9–12 months)		6‐Month follow‐up (age 12–15 months)	
	*N*	Mean (SD) or %	*N*	Mean (SD) or %	*N*	Mean (SD) or %
Choline (µmol/L)	60^a^	17.1 (3.5)	–	–	60	14.6 (3.6)
Betaine (µmol/L)	60^a^	85.1 (31.5)	–	–	60	96.7 (34.8)
TMAO (µmol/L)^b^	60^a^	1.8 (0.8, 3.8)	–	–	60	3.7 (2.2, 5.4)
Ferritin (µg/L)^c^	364	19.3 (18.2)	–	–	393	10.1 (11.9)
Zinc (µg/dl)^b^	364	53.0 (32.2, 82.7)	–	–	393	50.6 (31.1, 95.6)
CRP (mg/L)	364	8.6 (14.0)	–	–	393	6.7 (12.6)
AGP (g/L)	364	1.2 (0.6)	–	–	393	1.1 (0.6)
MDAT fine motor norm z‐score	393	1.0 (1.3)	–	–	386	0.5 (1.1)
MDAT gross motor norm z‐score	393	0.7 (0.8)	–	–	386	0.7 (1.2)
MDAT language norm z‐score	393	−0.002 (0.9)	–	–	387	0.2 (0.8)
MDAT personal–social norm z‐score	393	1.4 (0.9)	–	–	387	1.0 (1.1)
VPC novelty preference score (%)	162 (508 trials)	57 (17)	–	–	303 (950 trials)	60 (17)
VPC peak look length (ms)	164 (518 trials)	3836 (2342)	–	–	302 (931 trials)	3672 (2470)
IOWA response time (ms)	159 (3782 trials)	442.5 (188.1)	–	–	285 (7560 trials)	403.7 (178.2)
Elicited Imitation actions recalled	–	–	–	–	387	6.8 (3.5)
Length (cm)	400	66.8 (2.8)	399	70.2 (3.0)	400	73.7 (2.9)
Length‐for‐age z‐score	400	−0.9 (1.0)	399	−1.4 (1.1)	400	−1.1 (1.1)
Stunting (LAZ ≤ −2)	400	13.8%	399	28.1%	400	19.5%
Weight (kg)	400	7.7 (1.0)	399	8.6 (1.1)	400	9 (1.1)
Weight‐for‐age z‐score	400	−0.5 (1.1)	399	−0.5 (1.1)	400	−0.6 (1.1)
Underweight (WAZ ≤ −2)	400	8.3%	399	9.8%	400	11.0%
Weight‐for‐length z‐score	400	0.1 (1.1)	399	0.3 (1.1)	400	−0.2 (1.0)
Wasting (WLZ ≤ −2)	400	1.5%	399	1.3%	400	2.3%
Head circumference (cm)	400	42.1 (1.6)	398	43.9 (1.5)	400	45.6 (1.6)
Head circumference‐for‐age z‐score	400	−1.1 (1.1)	398	−0.9 (1.1)	400	−0.2 (1.2)
Low head circumference (HCAZ ≤ −2)	400	23.5%	398	17.3%	400	7.5%

Abbreviations: AGP, alpha(1)‐acid glycoprotein; CRP, C‐reactive protein; HCAZ, head circumference‐for‐age z‐score; IOWA, Infant Orienting with Attention task; LAZ, length‐for‐age z‐score; MDAT, Malawi Developmental Assessment Tool; VPC, visual paired comparison task; TMAO, trimethylamine N‐oxide; WAZ, weight‐for‐age z‐score; WLZ, weight‐for‐length z‐score.

^a^ Absolute concentrations were measured in a subset (*n* = 60) of the 400 children with semi‐quantitative metabolomics data.

^b^ Variable was skewed. Median (IQR) is presented.

^c^ Corrected for inflammation according to the BRINDA approach (Namaste et al., 2017).

Stunting was common, ranging from 14% at baseline to 29% at 6‐month follow‐up (Table 2). Because of the low prevalence of wasting (~1%–2%), it was not included in analysis of dichotomous outcomes. Both peak look length during the VPC familiarisation phase and response time during the IOWA task decreased over the study period. On average, children successfully recalled fewer than two of eight of the sequences in the elicited imitation task. Due to the uniform low number of sequences performed, this score was not used as a primary outcome.

### 3.2 Associations between plasma choline, growth and neurodevelopment

Plasma choline was not consistently associated with most measures of growth or development (Table 3). Plasma choline was positively associated with WLZ in a minimally adjusted cross‐sectional model, but the association was attenuated and no longer statistically significant in the fully adjusted model. There also was no association with conditional WLZ in either the minimally adjusted or fully adjusted predictive models. There was a weak but significant negative association between plasma choline and LAZ in the fully adjusted cross‐sectional model; however, there was no association with conditional LAZ in either of the predictive models. There was a positive, cross‐sectional association between plasma choline and IOWA response time, suggesting a slower response with higher plasma choline, and a negative, cross‐sectional association between plasma choline and peak look length, suggesting shorter peak looks with higher plasma choline. Both cross‐sectional associations remained significant after adjustment. However, no association was apparent in the predictive models. Baseline choline was negatively associated with fine motor normed z‐scores at 6‐month follow‐up in a fully adjusted predictive model, suggesting higher plasma choline predicted poorer fine motor development.

**Table 3:** Minimally and fully adjusted models of the association of plasma choline concentration (in SD units) with growth and development among participants of the Mazira Project (*n* = 400)

	Minimally adjusted^a^		Fully adjusted^b^	
	Estimate	95% CI	Estimate	95% CI
**Growth outcomes**				
*Cross‐sectional* c				
Length‐for‐age z‐score	−0.08	−0.17 to 0.004	**−0.09** *	**−0.17 to −0.01**
Weight‐for‐age z‐score	0.01	−0.07 to 0.10	0.01	−0.07 to 0.09
Weight‐for‐length z‐score	**0.08** *	**0.0003–0.17**	0.07	−0.03 to 0.16
Head circumference‐for‐age z‐score	−0.02	−0.11 to 0.06	−0.03	−0.12 to 0.05
Odds ratio for stunting (LAZ ≤ −2)	1.09	0.90–1.33	1.10	0.89–1.36
Odds ratio for underweight (WAZ ≤ −2)	1.00	0.78–1.29	1.04	0.80–1.36
Odds ratio for low head circumference (HCAZ ≤ −2)	0.89	0.71–1.11	0.90	0.71–1.14
*Predictive* d				
Conditional LAZ	0.04	−0.003 to 0.08	0.03	−0.02 to 0.07
Conditional WAZ	0.01	−0.03 to 0.06	−0.01	−0.06 to 0.03
Conditional WLZ	−0.01	−0.07 to 0.04	−0.03	−0.09 to 0.02
**Developmental outcomes**				
*Cross‐sectional* c				
MDAT fine motor norm z‐score	0.01	−0.06 to 0.08	0.01	−0.05 to 0.08
MDAT gross motor norm z‐score	−0.05	−0.12 to 0.03	−0.05	−0.12 to 0.03
MDAT language norm z‐score	−0.02	−0.08 to 0.04	−0.01	−0.07 to 0.04
MDAT personal–social norm z‐score	−0.01	−0.08 to 0.05	−0.01	−0.07 to 0.06
VPC novelty preference score (%)	0.5	−0.5 to 1.4	0.5	−0.4 to 1.4
VPC peak look length (ms)	**−203.5** *	**−366.2 to −40.7**	**−183.0** *	**−348.1 to −17.9**
IOWA response time (ms)	**8.33** *	**0.84–15.82**	**8.84** *	**1.66–16.03**
Elicited Imitation actions recalled	0.001	−0.28 to 0.28	0.06	−0.22 to 0.35
*Predictive* d				
MDAT fine motor norm z‐score	**−0.11** *	**−0.21 to −0.02**	**−0.13** *	**−0.22 to −0.04**
MDAT gross motor norm z‐score	−0.02	−0.14 to 0.10	−0.01	−0.13 to 0.11
MDAT language norm z‐score	−0.07	−0.14 to 0.01	−0.08	−0.16 to 0.001
MDAT personal–social norm z‐score	−0.04	−0.14 to 0.05	−0.04	−0.14 to 0.05
VPC novelty preference score (%)	0.8	−0.3 to 1.8	0.6	−0.6 to 1.7
VPC peak look length (ms)	78.5	−105.5 to 262.5	96.7	−77.8 to 271.2
IOWA response time (ms)	0.66	−8.76 to 10.08	0.46	−8.90 to 9.83
Elicited Imitation actions recalled	−0.21	−0.48 to 0.06	−0.18	−0.45 to 0.09

Abbreviations: HCAZ, head circumference‐for‐age z‐score; IOWA, Infant Orienting with Attention task; LAZ, length‐for‐age z‐score; MDAT, Malawi Developmental Assessment Tool; VPC, visual paired comparison task; WAZ, weight‐for‐age z‐score.

^a^ Adjusted for: time of last intake other than breast milk, water, or tea before blood draw, calendar month of blood draw, anthropometrist or developmental data assessor, group assignment, time point (for models including multiple time points). For developmental assessments, the child's mood, activity level and interaction with the assessor during tasks were included. For the elicited imitation actions recalled score, the ‘spontaneous actions' score was included. For eye‐tracking tasks, the eye‐tracking station was included. For the novelty preference score, the familiarisation time in seconds was included. For IOWA response time, condition was included.

^b^ Additionally adjusted for: child age, sex and birth order, baseline maternal age and education category, baseline household asset index and baseline food insecurity category. For growth outcomes, additional covariates included plasma leucine and zinc; plasma inflammatory markers (CRP and AGP); longitudinal prevalence of diarrhoea; and maternal height. For developmental outcomes, additional covariates included baseline plasma adjusted ferritin and child stimulation.

^c^ Continuous growth/developmental outcomes and dichotomous growth outcomes were assessed in generalised linear models and logistic regression models, respectively, with both time points, participant as independent unit, and robust standard errors.

^d^ Conditional growth measures and developmental outcomes were assessed in linear regression models, with baseline plasma choline as a predictor.

* *p* < 0.05.

### 3.3 Other choline metabolites

There were no significant associations of betaine, DMG or TMAO with growth indicators (Table 4). Among developmental outcomes, baseline TMAO was positively associated with peak look length and elicited imitation total actions score at 6‐month follow‐up (Table 4). Given that fish is a major dietary source of TMAO, fish consumption (any vs. none) at baseline was tested as a covariate in models of TMAO, with no significant changes to the results.

**Table 4:** Minimally adjusted^a^ regression models of the association of plasma betaine, dimethylglycine and trimethylamine N‐oxide concentrations (in SD units) with growth and development among participants of the Mazira Project (*n* = 400)

	Plasma betaine		Plasma DMG		Plasma TMAO	
	Estimate	95% CI	Estimate	95% CI	Estimate	95% CI
**Growth outcomes**						
*Cross‐sectional* b						
Length‐for‐age z‐score	−0.02	−0.11 to 0.06	−0.07	−0.26 to 0.12	−0.04	−0.11 to 0.04
Weight‐for‐age z‐score	0.01	−0.08 to 0.09	−0.15	−0.35 to 0.05	−0.04	−0.12 to 0.03
Weight‐for‐length z‐score	0.03	−0.05 to 0.11	−0.14	−0.34 to 0.05	−0.03	−0.11 to 0.04
Head circumference‐for‐age z‐score	0.02	−0.07 to 0.10	−0.12	−0.31 to 0.06	−0.04	−0.11 to 0.04
OR for stunting (LAZ ≤ −2)	0.95	0.78–1.16	0.86	0.57–1.29	0.99	0.83–1.17
OR for underweight (WAZ ≤ −2)	0.84	0.65–1.08	1.24	0.76–2.02	1.23	0.96–1.57
OR for low head circumference (HCAZ ≤ −2)	0.94	0.76–1.17	1.00	0.64–1.56	0.99	0.83–1.18
*Predictive* c						
Conditional LAZ	0.03	−0.01 to 0.07	0.07	−0.02 to 0.15	−0.02	−0.05 to 0.01
Conditional WAZ	0.02	−0.02 to 0.07	0.03	−0.06 to 0.13	−0.02	−0.05 to 0.02
Conditional WLZ	0.02	−0.04 to 0.07	−0.01	−0.12 to 0.11	−0.01	−0.05 to 0.03
**Developmental outcomes**						
*Cross‐sectional* b						
MDAT fine motor norm z‐score	−0.04	−0.11 to 0.03	0.13	−0.02 to 0.28	0.002	−0.06 to 0.07
MDAT gross motor norm z‐score	−0.05	−0.12 to 0.02	−0.04	−0.24 to 0.16	−0.01	−0.08 to 0.06
MDAT language norm z‐score	−0.004	−0.06 to 0.05	0.02	−0.10 to 0.15	−0.002	−0.05 to 0.05
MDAT personal–social norm z‐score	0.02	−0.05 to 0.08	−0.07	−0.22 to 0.08	0.003	−0.06 to 0.06
VPC novelty preference score (%)	0.6	−0.5 to 1.6	−0.4	−2.2 to 1.3	0.5	−0.5 to 1.5
VPC peak look length (ms)	44.8	−119.0 to 208.6	−2.4	−300.7 to 295.8	57.7	−86.2 to 201.6
IOWA response time (ms)	4.30	−4.21 to 12.80	2.36	−15.70 to 20.43	−4.14	−12.41 to 4.13
Elicited Imitation actions recalled	−0.03	−0.30 to 0.24	−0.47	−1.03 to 0.08	0.11	−0.20 to 0.41
*Predictive* c						
MDAT fine motor norm z‐score	−0.09	−0.19 to 0.002	0.02	−0.18 to 0.23	−0.06	−0.13 to 0.01
MDAT gross motor norm z‐score	−0.03	−0.15 to 0.10	0.17	−0.09 to 0.42	0.05	−0.04 to 0.14
MDAT language norm z‐score	−0.0002	−0.08 to 0.08	0.08	−0.08 to 0.25	−0.02	−0.08 to 0.03
MDAT personal–social norm z‐score	−0.02	−0.11 to 0.07	−0.12	−0.32 to 0.08	0.04	−0.03 to 0.11
VPC novelty preference score (%)	0.6	−0.8 to 2.0	−1.7	−3.8 to 0.5	−0.5	−1.4 to 0.4
VPC peak look length (ms)	71.8	−130.4 to 274.1	−72.9	−404.6 to 258.7	**231.7** *	**100.4–363.0**
IOWA response time (ms)	−2.62	−12.03 to 6.79	−6.05	−27.20 to 15.09	−4.66	−11.56 to 2.23
Elicited Imitation actions recalled	−0.16	−0.43 to 0.11	−0.17	−0.74 to 0.41	**0.23** *	**0.03–0.43**

Abbreviations: DMG, dimethylglycine; HCAZ, head circumference‐for‐age z‐score; IOWA, Infant Orienting with Attention task; LAZ, length‐for‐age z‐score; MDAT, Malawi Developmental Assessment Tool; TMAO, trimethylamine N‐oxide; VPC, visual paired comparison task; WAZ, weight‐for‐age z‐score.

^a^ Adjusted for: time of last intake other than breast milk, water, or tea before blood draw, calendar month of blood draw, anthropometrist or developmental data assessor, group assignment, time point (for models including multiple time points). For developmental assessments, the child's mood, activity level and interaction with the assessor during tasks were included. For the elicited imitation actions recalled score, the ‘spontaneous actions’ score was included. For eye‐tracking tasks, the eye‐tracking station was included. For the novelty preference score, the familiarisation time in seconds was included. For IOWA response time, condition was included.

^b^ Continuous growth/developmental outcomes and dichotomous growth outcomes were assessed in generalised linear models and logistic regression models, respectively, with both time points, participant as independent unit, and robust standard errors.

^c^ Conditional growth measures and developmental outcomes were assessed in linear regression models, with baseline plasma metabolite as a predictor.

* *p* < 0.05.

### 3.4 Effect modification analyses

Seven variables were tested as potential effect modifiers of the relationship between plasma choline and child growth outcomes. Out of 67 tests (Supporting Information: Table 2), 3 were significant at the 0.05 level (4.5%), which is near that expected by chance (5%; Supporting Information: Table 3). Fourteen potential effect modifiers were examined for their role in the relationship between plasma choline and the eight developmental outcomes. Out of 215 tests (Supporting Information: Table 2), 18 were significant at the 0.05 level (8.4%; Supporting Information: Table 3). No clear pattern was evident across effect modifiers.

## 4 DISCUSSION

Contrary to our hypothesis, plasma choline was not associated with most measures of child neurodevelopment and growth in this secondary analysis. The few significant associations suggested faster‐processing speed, poorer fine motor skills, slower orienting of attention and lower LAZ with higher plasma choline. Plasma betaine, DMG and TMAO were also not associated with most measures of development and growth.

These findings are important for understanding the null results of the Mazira Project randomised trial, in which the egg intervention was hypothesised to improve growth and development in part via improvement in choline status. The lack of effect in Malawi could have been due to a break in one or both links in the potential biologic pathway: eggs did not cause a change in plasma choline and/or plasma choline was not associated with growth and development in this context. We recently reported that plasma choline was not improved by the egg intervention trial in Malawi (Bragg et al., 2022), negating one portion of the hypothesised pathway to improved growth. In the present analysis, we found no connection between plasma choline and most measures of children's growth or neurodevelopment in this context of very low choline intake, negating the other portion of the hypothesised causal pathway. Of note, it is likely that other nutritional and non‐nutritional factors contributed to the null effects of the Mazira Trial, as choline status was only a partial mediator in a similar egg intervention trial in Ecuador (Iannotti, Lutter, Stewart, et al., 2017; Iannotti, Lutter, Waters, et al., 2017).

Choline is thought to affect growth and development through several mechanisms. First, choline is a precursor to phosphatidylcholine, a phospholipid required for the formation of cell membranes, as well as for fat absorption and transport. Phosphatidylcholine is also a major component of surfactant in the lung (Agassandian & Mallampalli, 2013) and the mucus layer in the gut (Bernhard et al., 1995) and is a carrier of DHA in plasma (Bernhard et al., 2018). Rodents without choline kinase beta, an enzyme on the pathway from choline to phosphatidylcholine, have altered endochondral bone growth with shortened forelimbs (Kular et al., 2015; Li et al., 2014). Additionally, maternal phosphatidylcholine intake affects offspring immune development in rodents (Lewis et al., 2016), which may contribute to optimal growth and neurodevelopment. Second, choline can be oxidised to betaine, a methyl donor involved in epigenetics. Epigenetic effects are thought to be crucial for choline's role in brain development (Zeisel, 2017), influencing neural progenitor cell mitosis and apoptosis (Albright et al., 1999; Wang et al., 2019). Third, choline may be converted to acetylcholine, a neurotransmitter involved in the encoding of new memories in the hippocampus. Acetylcholine is also a neuromodulator that influences neurogenesis and synapse formation (Haam & Yakel, 2017). In rodent knockout models, a lack of acetylcholine in the perinatal period reduced circulating levels of growth hormone and insulin‐like growth factor 1 (Lecomte et al., 2018). Finally, choline may be converted by gut microbiota to produce TMAO. Although TMAO is associated with atherosclerosis and inflammation in adults (Yang et al., 2019), its effects in young children are unclear. TMAO is also provided directly from fish consumption, separately from the choline metabolic pathway. The association of TMAO with developmental outcomes in this analysis may be related to fish consumption rather than choline metabolism; however, results were not changed when fish consumption was included as a covariate in statistical models.

Although there are few studies examining early postnatal choline status, especially in LMICs, previous studies have generally yielded positive or null results. Specific to growth, a cross‐sectional study of Malawian children found a significant positive association between serum choline and height‐for‐age z‐score and a significant negative association between serum choline and stunting (Semba et al., 2016). In Brazil, an observational study of children aged 6–24 months reported a negative association between stunting and urinary levels of betaine and DMG, suggesting altered choline metabolism among stunted children (Mayneris‐Perxachs et al., 2016). Related to neurodevelopment, provision of supplementary foods containing choline, along with other nutrients, to young children led to improvement in working memory in Guinea‐Bissau and locomotor skills in South Africa (Roberts et al., 2020; Smuts et al., 2019). In high‐income countries, early postnatal choline supplementation trials have yielded positive (Wozniak et al., 2015, 2020) or null (Andrew, Parr, Montague‐Johnson, Laler, Holmes, et al., 2018; Andrew, Parr, Montague‐Johnson, Laler, Qi, et al., 2018) results on neurodevelopmental outcomes. Observational studies in high‐income countries also note positive associations between choline or its metabolites and early neurodevelopment (Cheatham & Sheppard, 2015; Strain et al., 2013; Wiedeman, Chau, et al., 2018). Very few studies have reported a negative association of postnatal choline with growth or development. In a case–control study in the United States, young children with autism spectrum disorder had higher plasma choline and betaine concentrations, and those with Down syndrome had higher plasma choline and DMG concentrations, compared to typically developing children (Orozco et al., 2019).

We found inconsistent and mostly null associations of plasma choline with growth and developmental outcomes, with three of four significant associations suggesting poorer growth or development with higher plasma choline. The association of higher plasma choline with shorter peak looks, indicating faster‐processing speed, is interesting given the improvement in processing speed observed after prenatal choline supplementation found previously (Caudill et al., 2018); however, given the small effect size (−203.5 ms, or 0.09 SD, shorter peak look for each 1 SD lower plasma choline) and inconsistencies with other developmental outcomes, it is difficult to draw strong conclusions from this association. Our contrasting findings may be due to dietary and sociodemographic factors unique to the study population. Choline's status may have been lower in this setting than in previous reports. Because plasma choline concentration decreases over the first years of life (Ilcol et al., 2005), it is difficult to compare plasma choline status across studies with various age ranges. However, choline intake was far below recommendations in this study population, even with the egg intervention. At baseline, the mean estimated usual choline intake per day was 102 (SE 1) mg, far below the AI for infants 7–12 months (150 mg/d; Caswell et al., 2021). Although egg and choline intakes were increased due to the intervention at 6‐month follow‐up, ~97% of children in the intervention group and 100% of children in the control group still had estimated intakes below recommendations (Caswell et al., 2021; Stewart et al., 2019). Much of choline's effect on neurodevelopment is thought to be through conversion to betaine. However, when body stores are low, choline oxidation to betaine is limited (Li & Vance, 2008). Perhaps there is a threshold effect, such that at very low intakes, choline is not converted to betaine at a rate that affects epigenetic control of neurodevelopment. Alternatively, perhaps in the context of undernutrition, betaine is used in its other role as an osmolyte assisting in cell volume regulation (Lever & Slow, 2010), rather than as a methyl donor. Genetics may also play a role, given the existence of common SNPs which influence choline metabolism (Ganz et al., 2017); however, genetic data were not collected for this study. Additionally, there may have been a greater number of health and environmental factors that constrained growth and neurodevelopment in this low‐income setting, especially compared to studies in high‐income countries. In addition to nutrition, child growth and development are affected by a range of factors including maternal health, infectious disease and environmental toxins (Danaei et al., 2016; Stewart et al., 2013; Walker et al., 2011). This study population had high rates of anaemia and inflammation, with low maternal education and socioeconomic status.

These findings could also be due to limitations of plasma choline as a marker of choline status. Plasma choline is an imperfect biomarker which may not reflect small to moderate changes in intake (Abratte et al., 2009). Currently, sensitive and specific markers of choline status are lacking, although this is an active area of research (Zeisel, 2018). We included several related metabolites (betaine, DMG and TMAO) as a means to investigate choline status, but other metabolites, such as phosphatidylcholine, were not included. Although phosphatidylcholine may be important for cell growth, its measurement is affected by fat transport and metabolism. Additionally, plasma concentrations of these nutrients may not reflect concentrations in the brain, which may be more closely related to neurodevelopment. Future studies with validated biomarkers of choline status may help uncover the relationship between choline, growth and development. If this is a true null association, additional interventions are needed to improve children's growth and development in this setting.

This analysis had several strengths. First, the large sample size allowed for detection of small associations. Second, measurement of plasma choline and neurodevelopment at two time points, and anthropometry at three time points, allowed for longitudinal analysis. The current analysis builds on prior studies by including predictive, as well as cross‐sectional, pre‐specified analyses. Third, several types of anthropometric outcomes were included, based on best practices. Additionally, because plasma choline was on the hypothesised causal pathway of the main trial, developmental assessments were chosen specifically to match domains that choline is likely to influence (memory and attention). The study also included eye‐tracking measures which may be more sensitive to small changes in development than measures that assess the acquisition of developmental milestones (Aslin, 2007).

This study also had weaknesses. As a secondary analysis, this observational study used data from a trial that was not specifically designed to test this research question. Because of its observational design, there is risk for bias and confounding; however, this risk was minimised with the use of blinded outcome assessors, objective plasma measures, a prospective design with high rates of study follow‐up and statistical adjustment for a range of covariates. These findings are correlations, and statements of causation cannot be made. Due to the multitude of statistical tests, some findings may be due to chance, although it is suggestive that three of four significant findings are in the direction of poorer growth and development with higher plasma choline. Also, given the inconsistencies in findings relating to choline and growth even within Malawi, the generalisability of this study is likely limited to LMIC settings with similar anthropometric, developmental, dietary and socioeconomic characteristics.

## 5 CONCLUSION

In this observational study, plasma choline and its metabolites were not related to most measures of growth or development among a sample of young Malawian children. More information is needed on the role of choline for child growth and development in varied contexts; future studies will require validated biomarkers and rigorous study designs. Adequate intake of choline should still be recommended for young children, as it is an essential nutrient with multiple roles throughout the body.

## AUTHOR CONTRIBUTIONS

Christine P. Stewart, Kenneth M. Maleta and Elizabeth L. Prado designed the Mazira Project randomised trial. Bess L. Caswell conducted the study, and Matthews George collected the data. Lisa M. Oakes provided guidance on the eye‐tracking and elicited imitation assessments; Lisa M. Oakes, Michaela C. DeBolt and Aaron G. Beckner aided in analysis of eye‐tracking data; and Aaron G. Beckner coded the peak look variable. Brian J. Bennett performed the quantitative lab analyses. Megan G. Bragg and Charles D. Arnold conducted the data analysis, and Megan G. Bragg wrote the manuscript. All authors read and approved the final manuscript.

## CONFLICT OF INTEREST

The authors declare no conflict of interest.

## Supporting information

Supporting information.

## Data Availability

Data from the Mazira Project are available on our OSF page: https://osf.io/vfrg7/.
