# Supplementary material for: The association between plasma choline, growth and neurodevelopment among Malawian children aged 6–15 months enroled in an egg intervention trial
Source: Matern Child Nutr. 2022 Dec 25;19(2):e13471. doi: 10.1111/mcn.13471 (PMC10019050; doi:10.1111/mcn.13471)
Supplement: Supplementary file 1 — Supporting information. [file MCN-19-e13471-s001.docx]

Supplemental Material

The association between plasma choline, growth, and neurodevelopment among Malawian children age 6-15 months enrolled in an egg intervention trial

Authors: Megan G Bragg^1^; Elizabeth L Prado^1^; Bess L Caswell^2^; Charles D Arnold^1^; Matthews George^3^; Lisa M Oakes^4^; Aaron G Beckner^4^; Michaela C DeBolt^4^; Brian J Bennett^2^; Kenneth M Maleta^3^; Christine P Stewart^1^

^1^Department of Nutrition, University of California Davis, Davis, CA
^2^USDA Western Human Nutrition Research Center, Davis, CA

^3^School of Public Health and Family Medicine, Kamuzu University of Health Sciences, Blantyre, Malawi

^5^Center for Mind and Brain, University of California Davis, Davis, CA

Supplemental Figure 1 – Plasma choline (A), betaine (B), dimethylglycine (C), and trimethylamine N-oxide (D) by child age among children enrolled in the Mazira Project (n=400)

Supplemental Table 1 – Baseline characteristics of children enrolled in the Mazira Project and included vs excluded from the current secondary analysis

Supplemental Table 2 – Stratified results from significant effect modification analyses of the relationships between plasma choline, growth, and development among participants of the Mazira Project

Supplemental Table 3 – Interaction tests from effect modification analyses of the relationship between plasma choline, growth, and development among participants of the Mazira Project

Supplemental Figure 1 – Plasma choline (A), betaine (B), dimethylglycine (C), and trimethylamine N-oxide (D) by child age among children enrolled in the Mazira Project (n=400)^a^

1. Choline


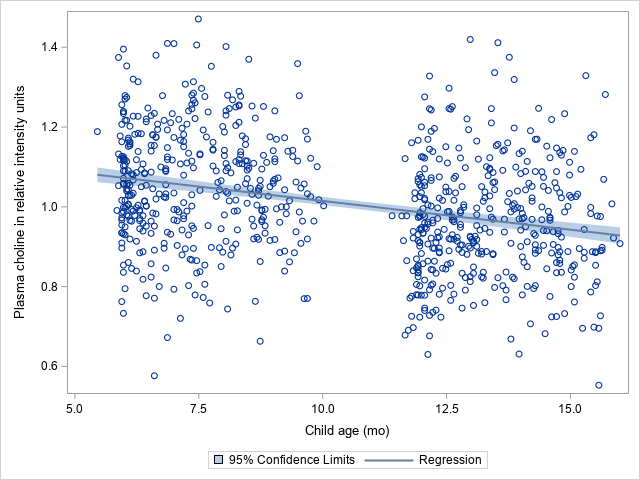


1. Betaine


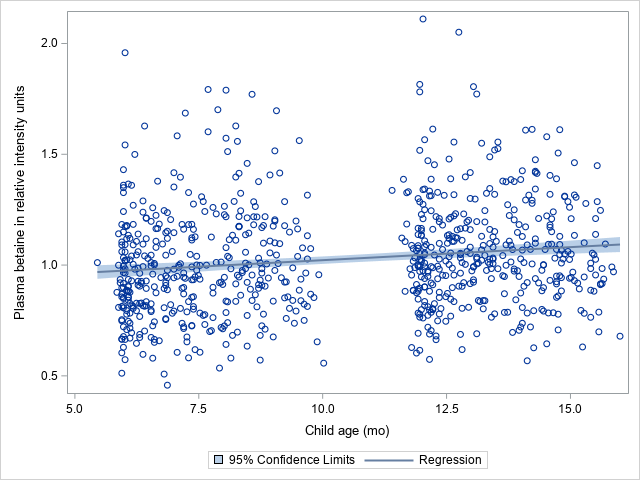


1. Dimethylglycine


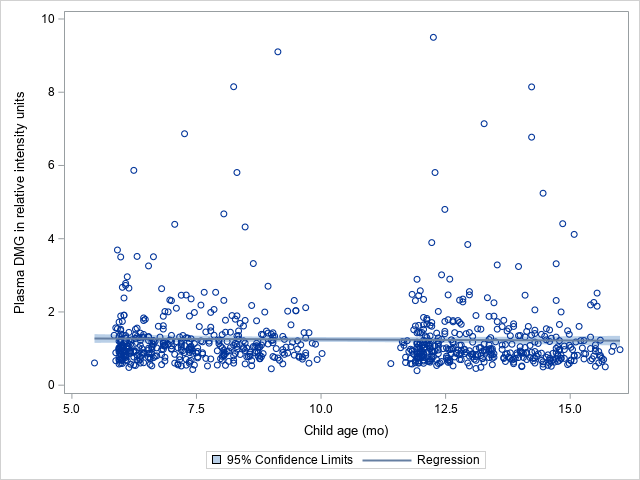


1. Trimethylamine N-oxide


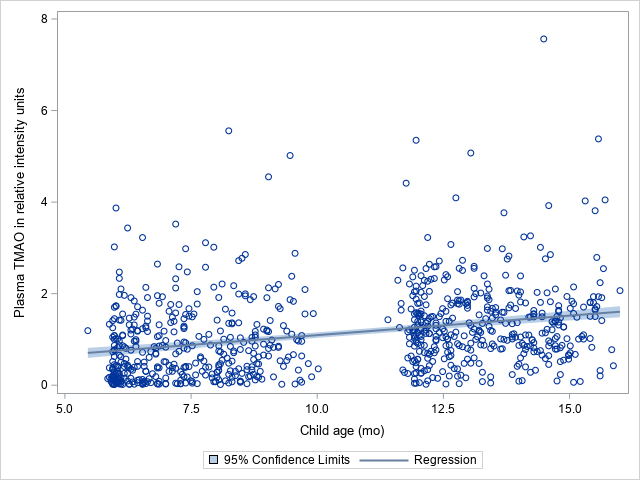


^a^ Solid lines are unadjusted regression lines; gray shading is the 95% confidence interval.

Supplemental Table 1 – Baseline characteristics of children enrolled in the Mazira Project and included vs excluded from the current secondary analysis

|  | Included (n=400) | Excluded (n= 260)^a^ | p value |
| --- | --- | --- | --- |
|  | Mean (SD) or (%) | Mean (SD) or (%) |  |
| *Child and maternal characteristics* |  |  |  |
| Child age (mo) | 7.4 (1.2) | 7.4 (1.2) | 0.849 |
| Male | 53.3 | 49.2 | 0.313 |
| First born | 27.8 | 27.3 | 0.886 |
| Animal source food consumption^b^ |  |  |  |
| Consumed dairy | 9.8 | 6.5 | 0.145 |
| Consumed meat | 2.5 | 0.8 | 0.103 |
| Consumed egg | 3.8 | 4.6 | 0.588 |
| Consumed fish | 23.6 | 32.7 | **0.010*** |
| Any breastmilk | 99.7 | 100.0 | 0.419 |
| Anemia prevalence (Hgb < 11 g/dL) | 62.2 | 57.8 | 0.320 |
| Malaria prevalence | 14.5 | 8.6 | 0.042 |
| Longitudinal prevalence of diarrhea | 0.1 (0.1) | 0.1 (0.1) | 0.345 |
| Maternal age (y) | 26.0 (6.8) | 25.9 (6.7) | 0.857 |
| Maternal BMI (kg/m^2^) | 21.8 (3.0) | 21.9 (3.0) | 0.445 |
| Maternal height (cm) | 156.9 (5.2) | 156.2 (5.3) | 0.110 |
| Mother completed primary school | 20.5 | 19.1 | 0.656 |
|  |  |  |  |
| *Household characteristics* |  |  |  |
| Number of household members | 6.0 (2.6) | 5.7 (2.7) | 0.242 |
| Moderate to severe food insecurity^c^ | 78.0 | 77.7 | 0.926 |
| Owns latrine | 96.5 | 96.3 | 0.918 |
| Owns cows | 2.5 | 3.7 | 0.399 |
| Owns goats | 20.0 | 17.3 | 0.388 |
| Owns chickens | 33.3 | 31.0 | 0.552 |
| HOME Inventory score | 24.0 (3.5) | 24.4 (3.5) | 0.207 |
| Family Care Indicators score^d^ | 7.3 (3.6) | 7.3 (3.6) | 0.860 |

^a^ Children enrolled in the trial were excluded from this secondary analysis if they were missing one or both blood samples (n=147) or were not randomly selected for biochemical analysis (n=113)

^b^ As reported by caregiver on a 24-hour dietary recall

^c^ As defined by the Household Food Insecurity Access scale (Coates et al., 2007)

^d^ Measured at 6 month follow up only

*p<0.05

Supplemental Table 2 – Stratified results from significant effect modification analyses^a^ of the relationships between plasma choline, growth, and development among participants of the Mazira Project

|  | Minimally Adjusted | |  |
| --- | --- | --- | --- |
|  | Estimate^b^ | 95% CI | p for interaction |
| Growth outcomes |  |  |  |
| Length-for-age z-score |  |  |  |
| *Cross-sectional^c^* |  |  |  |
| Housing and asset index (quintiles 2-5) | -0.03 | -0.13, 0.06 | **0.001*** |
| Housing and asset index (quintile 1) | **-0.38*** | **-0.55, -0.21** |  |
| *Predictive^d^* |  |  |  |
| Child sex (male) | -0.01 | -0.07, 0.05 | **0.030*** |
| Child sex (female) | **0.08*** | **0.02, 0.14** |  |
|  |  |  |  |
| Weight-for-length z-score |  |  |  |
| *Cross-sectional* |  |  |  |
| Longitudinal prevalence of diarrhea (below median) | **0.17*** | **0.06, 0.29** | **0.038*** |
| Longitudinal prevalence of diarrhea (above median) | -0.003 | -0.12, 0.11 |  |
|  |  |  |  |
| Developmental outcomes |  |  |  |
| MDAT fine motor norm z-score |  |  |  |
| *Cross sectional*^c^ |  |  |  |
| Housing and asset index (quintiles 2-5) | 0.05 | -0.03, 0.13 | **0.014*** |
| Housing and asset index (quintile 1) | -0.15 | -0.31, 0.02 |  |
|  |  |  |  |
| MDAT gross motor norm z-score |  |  |  |
| *Predictive^d^* |  |  |  |
| FCI score (above median) | **-0.21*** | **-0.40, -0.02** | **0.015*** |
| FCI score (below median) | 0.13 | -0.03, 0.29 |  |
| Baseline gross motor z-score (above median) | 0.17 | -0.01, 0.35 | **0.029*** |
| Baseline gross motor z-score (below median) | **-0.18*** | **-0.35, -0.01** |  |
|  |  |  |  |
| MDAT personal social norm z-score |  |  |  |
| *Cross sectional* |  |  |  |
| Housing and asset index (quintiles 2-5) | 0.03 | -0.04, 0.10 | **0.011*** |
| Housing and asset index (quintile 1) | **-0.16*** | **-0.31, -0.02*** |  |
| Visit code (baseline) | 0.06 | -0.04, 0.15 | **0.014*** |
| Visit code (6 month follow up) | -0.09 | -0.19, 0.01 |  |
| *Predictive* |  |  |  |
| Baseline DHA level (above median) | **-0.15*** | **-0.29, -0.01** | **0.038*** |
| Baseline DHA level (below median) | 0.04 | -0.08, 0.17 |  |
|  |  |  |  |
| MDAT language norm z-score |  |  |  |
| *Cross sectional* |  |  |  |
| Child sex (male) | -0.08 | -0.17, 0.01 | **0.045*** |
| Child sex (female) | 0.04 | -0.04, 0.12 |  |
| Group assignment (Egg intervention) | 0.04 | -0.04, 0.12 | **0.017*** |
| Group assignment (Control) | **-0.10*** | **-0.19, -0.01** |  |
| Food insecurity score (mild to none) | **-0.12*** | **-0.25, -0.004** | **0.019*** |
| Food insecurity score (moderate to severe) | 0.004 | -0.06, 0.07 |  |
| *Predictive* |  |  |  |
| FCI score (above median) | **-0.16*** | **-0.29, -0.04** | **0.041*** |
| FCI score (below median) | 0.01 | -0.10, 0.11 |  |
|  |  |  |  |
| VPC novelty preference (%) |  |  |  |
| *Cross-sectional* |  |  |  |
| Baseline DHA level (above median) | **-2.7*** | **-5.5, -0.01** | **0.019*** |
| Baseline DHA level (below median) | **3.9*** | **1.0, 6.7** |  |
|  |  |  |  |
| IOWA response time (ms) |  |  |  |
| *Cross-sectional* |  |  |  |
| Birth order (not firstborn) | 3.73 | -5.70, 13.17 | **0.038*** |
| Birth order (firstborn) | **18.23*** | **6.77, 29.69** |  |
| Maternal age (over 20y) | 2.63 | -6.55, 11.81 | **0.005*** |
| Maternal age (20y or younger) | **16.50*** | **6.38, 26.62** |  |
| Baseline IOWA response time (above median) | 2.54 | -6.77, 11.86 | **0.049*** |
| Baseline IOWA response time (below median) | -3.79 | -9.73, 2.15 |  |
| *Predictive* |  |  |  |
| Group assignment (Egg intervention) | 10.04 | -1.60, 21.67 | **0.014*** |
| Group assignment (Control) | **-13.31*** | **-26.09, -0.54** |  |
|  |  |  |  |
| Elicited Imitation actions recalled |  |  |  |
| *Cross-sectional, 6 month follow up* *only* |  |  |  |
| Baseline MDAT score (above median) | -0.22 | -0.64, 0.20 | **0.038*** |
| Baseline MDAT score (below median) | 0.24 | -0.17, 0.65 |  |
| *Predictive* |  |  |  |
| FCI score (above median) | **-0.55*** | **-0.93, -0.16** | **0.032*** |
| FCI score (below median) | 0.05 | -0.35, 0.44 |  |
| Housing and asset index (quintiles 2-5) | -0.10 | -0.40, 0.20 | **0.031*** |
| Housing and asset index (quintile 1) | -0.75 | -1.60, 0.10 |  |

^IOWA – Infant Orienting with Attention task; MDAT – Malawi Developmental Assessment Tool; VPC – visual paired comparison task^

^a^ Potential effect modifiers included child sex, baseline LAZ or stunting status, baseline maternal age, baseline housing and asset index, and time point of data collection (for models with both time points). Additionally, for growth: longitudinal prevalence of diarrhea. Additionally, for development: group assignment, birth order, maternal education, baseline household food insecurity, baseline developmental score, HOME score below median, FCI score below median, plasma ferritin and plasma DHA. Each modifier was made dichotomous. Stratified results are only presented for analyses in which the p-for-interaction was statistically significant (p<0.05).

^b^ Estimates are the mean difference in the growth or developmental outcome variable per 1 standard deviation difference in plasma choline.
^c^ Continuous growth/developmental outcomes and dichotomous growth outcomes were assessed in generalized linear models and logistic regression models, respectively, with both time points, participant as independent unit, and robust standard errors.

^d^ Conditional growth measures and developmental outcomes were assessed in linear regression models, with baseline plasma metabolite as a predictor.

*p<0.05

Supplemental Table 3 – Interaction tests from effect modification analyses^a^ of the relationship between plasma choline, growth, and development among participants of the Mazira Project

|  | p for interaction |
| --- | --- |
| Growth outcomes |  |
| Length-for-age z-score |  |
| *Cross-sectional^b^* |  |
| Child sex | 0.254 |
| Group assignment | 0.478 |
| Housing and asset index | **0.001*** |
| Maternal education | 0.704 |
| Longitudinal prevalence of diarrhea | 0.868 |
| Stunting status at baseline | 0.823 |
| Study visit | 0.844 |
| *Cross-sectional: Stunted (LAZ ≤ -2)^b^* |  |
| Child sex | 0.078 |
| Group assignment | 0.143 |
| Housing and asset index | 0.174 |
| Maternal education | 0.381 |
| Longitudinal prevalence of diarrhea | 0.618 |
| Stunting status at baseline | 0.382 |
| Study visit | 0.609 |
| *Predictive^c^* |  |
| Child sex | **0.038*** |
| Group assignment | 0.716 |
| Housing and asset index | 0.605 |
| Maternal education | 0.707 |
| Longitudinal prevalence of diarrhea | 0.957 |
| Stunting status at baseline | 0.606 |
|  |  |
| Weight-for-age z-score |  |
| *Cross-sectional* |  |
| Child sex | 0.403 |
| Group assignment | 0.450 |
| Housing and asset index | 0.377 |
| Maternal education | 0.268 |
| Longitudinal prevalence of diarrhea | 0.067 |
| Underweight status at baseline | 0.925 |
| Study visit | 0.812 |
| *Cross-sectional: Underweight (WAZ ≤ -2)* |  |
| Child sex | 0.954 |
| Group assignment | 0.054 |
| Housing and asset index | 0.598 |
| Maternal education | 0.694 |
| Longitudinal prevalence of diarrhea | 0.521 |
| Underweight status at baseline | 0.734 |
| Study visit | 0.448 |
| *Predictive* |  |
| Child sex | 0.195 |
| Group assignment | 0.684 |
| Housing and asset index | 0.895 |
| Maternal education | 0.896 |
| Longitudinal prevalence of diarrhea | 0.260 |
| Underweight status at baseline | 0.876 |
|  |  |
| Weight-for-length z-score |  |
| *Cross-sectional* |  |
| Child sex | 0.789 |
| Group assignment | 0.669 |
| Housing and asset index | 0.547 |
| Maternal education | 0.203 |
| Longitudinal prevalence of diarrhea | **0.030*** |
| Wasting status at baseline | 0.488 |
| Study visit | 0.918 |
| *Predictive* |  |
| Child sex | 0.551 |
| Group assignment | 0.806 |
| Housing and asset index | 0.608 |
| Maternal education | 0.724 |
| Longitudinal prevalence of diarrhea | 0.071 |
| Wasting status at baseline | 0.491 |
|  |  |
| Head circumference-for-age z-score |  |
| *Cross-sectional* |  |
| Child sex | 0.869 |
| Group assignment | 0.907 |
| Housing and asset index | 0.634 |
| Maternal education | 0.635 |
| Longitudinal prevalence of diarrhea | 0.327 |
| Low head circumference status at baseline | 0.189 |
| Study visit | 0.390 |
| *Cross-sectional: Low head circumference (HCAZ ≤ -2)* |  |
| Child sex | 0.690 |
| Group assignment | 0.600 |
| Housing and asset index | 0.095 |
| Maternal education | 0.079 |
| Longitudinal prevalence of diarrhea | 0.217 |
| Low head circumference status at baseline | 0.427 |
| Study visit | 0.939 |
|  |  |
| Developmental outcomes |  |
| MDAT fine motor norm z-score |  |
| *Cross sectional^b^* |  |
| Visit code (baseline vs 6 mo follow up) | 0.354 |
| Group (intervention vs control) | 0.843 |
| Child sex (male vs female) | 0.290 |
| Birth order (first vs not first) | 0.439 |
| Baseline DHA (below vs above median) | 0.065 |
| Baseline adjusted ferritin (below vs above <12 µg/L) | 0.914 |
| Baseline LAZ (below vs above <-1) | 0.086 |
| Baseline fine motor norm z-score (below vs above median) | 0.985 |
| Maternal age (below vs above 20y) | 0.455 |
| Maternal education (incomplete primary vs primary or greater) | 0.653 |
| FCI score (below vs above median) | 0.751 |
| HOME score (below vs above median) | 0.620 |
| Household food insecurity score (none/mild vs moderate/severe) | 0.804 |
| Household asset index quintile (quintile 1 vs 2-5) | **0.014*** |
| *Predictive^c^* |  |
| Group (intervention vs control) | 0.068 |
| Child sex (male vs female) | 0.217 |
| Birth order (first vs not first) | 0.753 |
| Baseline DHA (below vs above median) | 0.477 |
| Baseline adjusted ferritin (below vs above <12 µg/L) | 0.939 |
| Baseline LAZ (below vs above <-1) | 0.901 |
| Baseline fine motor norm z-score (below vs above median) | 0.722 |
| Maternal age (below vs above 20y) | 0.401 |
| Maternal education (incomplete primary vs primary or greater) | 0.654 |
| FCI score (below vs above median) | 0.092 |
| HOME score (below vs above median) | 0.257 |
| Household food insecurity score (none/mild vs moderate/severe) | 0.553 |
| Household asset index quintile (quintile 1 vs 2-5) | 0.466 |
|  |  |
| MDAT gross motor norm z-score |  |
| *Cross sectional* |  |
| Visit code (baseline vs 6 mo follow up) | 0.898 |
| Group (intervention vs control) | 0.874 |
| Child sex (male vs female) | 0.831 |
| Birth order (first vs not first) | 0.113 |
| Baseline DHA (below vs above median) | 0.244 |
| Baseline adjusted ferritin (below vs above <12 µg/L) | 0.737 |
| Baseline LAZ (below vs above <-1) | 0.900 |
| Baseline gross motor norm z-score (below vs above median) | 0.758 |
| Maternal age (below vs above 20y) | 0.804 |
| Maternal education (incomplete primary vs primary or greater) | 0.862 |
| FCI score (below vs above median) | 0.337 |
| HOME score (below vs above median) | 0.625 |
| Household food insecurity score (none/mild vs moderate/severe) | 0.760 |
| Household asset index quintile (quintile 1 vs 2-5) | 0.146 |
| *Predictive* |  |
| Group (intervention vs control) | 0.253 |
| Child sex (male vs female) | 0.463 |
| Birth order (first vs not first) | 0.624 |
| Baseline DHA (below vs above median) | 0.715 |
| Baseline adjusted ferritin (below vs above <12 µg/L) | 0.643 |
| Baseline LAZ (below vs above <-1) | 0.502 |
| Baseline gross motor norm z-score (below vs above median) | **0.029*** |
| Maternal age (below vs above 20y) | 0.436 |
| Maternal education (incomplete primary vs primary or greater) | 0.664 |
| FCI score (below vs above median) | **0.015** |
| HOME score (below vs above median) | 0.851 |
| Household food insecurity score (none/mild vs moderate/severe) | 0.903 |
| Household asset index quintile (quintile 1 vs 2-5) | 0.156 |
|  |  |
| MDAT personal social norm z-score |  |
| *Cross sectional* |  |
| Visit code (baseline vs 6 mo follow up) | **0.014*** |
| Group (intervention vs control) | 0.616 |
| Child sex (male vs female) | 0.572 |
| Birth order (first vs not first) | 0.258 |
| Baseline DHA (below vs above median) | 0.544 |
| Baseline adjusted ferritin (below vs above <12 µg/L) | 0.796 |
| Baseline LAZ (below vs above <-1) | 0.685 |
| Baseline personal social norm z-score (below vs above median) | 0.481 |
| Maternal age (below vs above 20y) | 0.730 |
| Maternal education (incomplete primary vs primary or greater) | 0.503 |
| FCI score (below vs above median) | 0.598 |
| HOME score (below vs above median) | 0.712 |
| Household food insecurity score (none/mild vs moderate/severe) | 0.425 |
| Household asset index quintile (quintile 1 vs 2-5) | **0.011*** |
| *Predictive* |  |
| Group (intervention vs control) | 0.943 |
| Child sex (male vs female) | 0.921 |
| Birth order (first vs not first) | 0.554 |
| Baseline DHA (below vs above median) | **0.038*** |
| Baseline adjusted ferritin (below vs above <12 µg/L) | 0.074 |
| Baseline LAZ (below vs above <-1) | 0.129 |
| Baseline personal social norm z-score (below vs above median) | 0.131 |
| Maternal age (below vs above 20y) | 0.324 |
| Maternal education (incomplete primary vs primary or greater) | 0.363 |
| FCI score (below vs above median) | 0.142 |
| HOME score (below vs above median) | 0.087 |
| Household food insecurity score (none/mild vs moderate/severe) | 0.275 |
| Household asset index quintile (quintile 1 vs 2-5) | 0.298 |
|  |  |
| MDAT language norm z-score |  |
| *Cross sectional* |  |
| Visit code (baseline vs 6 mo follow up) | 0.848 |
| Group (intervention vs control) | **0.017*** |
| Child sex (male vs female) | **0.045*** |
| Birth order (first vs not first) | 0.897 |
| Baseline DHA (below vs above median) | 0.606 |
| Baseline adjusted ferritin (below vs above <12 µg/L) | 0.958 |
| Baseline LAZ (below vs above <-1) | 0.373 |
| Baseline language norm z-score (below vs above median) | 0.122 |
| Maternal age (below vs above 20y) | 0.131 |
| Maternal education (incomplete primary vs primary or greater) | 0.488 |
| FCI score (below vs above median) | 0.120 |
| HOME score (below vs above median) | 0.500 |
| Household food insecurity score (none/mild vs moderate/severe) | **0.019*** |
| Household asset index quintile (quintile 1 vs 2-5) | 0.786 |
| *Predictive* |  |
| Group (intervention vs control) | 0.918 |
| Child sex (male vs female) | 0.855 |
| Birth order (first vs not first) | 0.963 |
| Baseline DHA (below vs above median) | 0.453 |
| Baseline adjusted ferritin (below vs above <12 µg/L) | 0.075 |
| Baseline LAZ (below vs above <-1) | 0.597 |
| Baseline language norm z-score (below vs above median) | 0.179 |
| Maternal age (below vs above 20y) | 0.199 |
| Maternal education (incomplete primary vs primary or greater) | 0.691 |
| FCI score (below vs above median) | **0.041*** |
| HOME score (below vs above median) | 0.548 |
| Household food insecurity score (none/mild vs moderate/severe) | 0.476 |
| Household asset index quintile (quintile 1 vs 2-5) | 0.762 |
|  |  |
| Novelty preference score |  |
| *Cross sectional* |  |
| Visit code (baseline vs 6 mo follow up) | 0.513 |
| Group (intervention vs control) | 0.351 |
| Child sex (male vs female) | 0.553 |
| Birth order (first vs not first) | 0.979 |
| Baseline DHA (below vs above median) | **0.019*** |
| Baseline adjusted ferritin (below vs above <12 µg/L) | 0.899 |
| Baseline LAZ (below vs above <-1) | 0.352 |
| Baseline novelty preference score (below vs above median) | 0.652 |
| Maternal age (below vs above 20y) | 0.810 |
| Maternal education (incomplete primary vs primary or greater) | 0.362 |
| FCI score (below vs above median) | 0.936 |
| HOME score (below vs above median) | 0.836 |
| Household food insecurity score (none/mild vs moderate/severe) | 0.862 |
| Household asset index quintile (quintile 1 vs 2-5) | 0.690 |
| *Predictive* |  |
| Group (intervention vs control) | 0.732 |
| Child sex (male vs female) | 0.072 |
| Birth order (first vs not first) | 0.793 |
| Baseline DHA (below vs above median) | 0.633 |
| Baseline adjusted ferritin (below vs above <12 µg/L) | 0.366 |
| Baseline LAZ (below vs above <-1) | 0.336 |
| Baseline novelty preference score (below vs above median) | 0.236 |
| Maternal age (below vs above 20y) | 0.401 |
| Maternal education (incomplete primary vs primary or greater) | 0.709 |
| FCI score (below vs above median) | 0.779 |
| HOME score (below vs above median) | 0.648 |
| Household food insecurity score (none/mild vs moderate/severe) | 0.962 |
| Household asset index quintile (quintile 1 vs 2-5) | 0.582 |
|  |  |
| VPC peak look length |  |
| *Cross sectional* |  |
| Visit code (baseline vs 6 mo follow up) | 0.957 |
| Group (intervention vs control) | 0.499 |
| Child sex (male vs female) | 0.498 |
| Birth order (first vs not first) | 0.336 |
| Baseline DHA (below vs above median) | 0.395* |
| Baseline adjusted ferritin (below vs above <12 µg/L) | 0.636 |
| Baseline LAZ (below vs above <-1) | 0.732 |
| Baseline peak look length (below vs above median) | 0.666 |
| Maternal age (below vs above 20y) | 0.172 |
| Maternal education (incomplete primary vs primary or greater) | 0.458 |
| FCI score (below vs above median) | 0.632 |
| HOME score (below vs above median) | 0.501 |
| Household food insecurity score (none/mild vs moderate/severe) | 0.435 |
| Household asset index quintile (quintile 1 vs 2-5) | 0.369 |
| *Predictive* |  |
| Group (intervention vs control) | 0.757 |
| Child sex (male vs female) | 0.969 |
| Birth order (first vs not first) | 0.116 |
| Baseline DHA (below vs above median) | 0.843 |
| Baseline adjusted ferritin (below vs above <12 µg/L) | 0.672 |
| Baseline LAZ (below vs above <-1) | 0.241 |
| Baseline peak look length (below vs above median) | 0.801 |
| Maternal age (below vs above 20y) | 0.196 |
| Maternal education (incomplete primary vs primary or greater) | 0.898 |
| FCI score (below vs above median) | 0.118 |
| HOME score (below vs above median) | 0.544 |
| Household food insecurity score (none/mild vs moderate/severe) | 0.197 |
| Household asset index quintile (quintile 1 vs 2-5) | 0.707 |
|  |  |
| IOWA response time |  |
| *Cross sectional* |  |
| Visit code (baseline vs 6 mo follow up) | 0.475 |
| Group (intervention vs control) | 0.825 |
| Child sex (male vs female) | 0.670 |
| Birth order (first vs not first) | 0.038 |
| Baseline DHA (below vs above median) | 0.675 |
| Baseline adjusted ferritin (below vs above <12 µg/L) | 0.514 |
| Baseline LAZ (below vs above <-1) | 0.084 |
| Baseline response time (below vs above median) | **0.049*** |
| Maternal age (below vs above 20y) | **0.005*** |
| Maternal education (incomplete primary vs primary or greater) | 0.285 |
| FCI score (below vs above median) | 0.953 |
| HOME score (below vs above median) | 0.394 |
| Household food insecurity score (none/mild vs moderate/severe) | 0.807 |
| Household asset index quintile (quintile 1 vs 2-5) | 0.760 |
| *Predictive* |  |
| Group (intervention vs control) | **0.014*** |
| Child sex (male vs female) | 0.264 |
| Birth order (first vs not first) | 0.920 |
| Baseline DHA (below vs above median) | 0.501 |
| Baseline adjusted ferritin (below vs above <12 µg/L) | 0.651 |
| Baseline LAZ (below vs above <-1) | 0.243 |
| Baseline response time (below vs above median) | 0.469 |
| Maternal age (below vs above 20y) | 0.821 |
| Maternal education (incomplete primary vs primary or greater) | 0.893 |
| FCI score (below vs above median) | 0.485 |
| HOME score (below vs above median) | 0.958 |
| Household food insecurity score (none/mild vs moderate/severe) | 0.849 |
| Household asset index quintile (quintile 1 vs 2-5) | 0.714 |
|  |  |
| Elicited imitations total actions recalled |  |
| *Cross sectional model, 6 month follow up only* |  |
| Group (intervention vs control) | 0.701 |
| Child sex (male vs female) | 0.229 |
| Birth order (first vs not first) | 0.813 |
| Baseline DHA (below vs above median) | 0.451 |
| Baseline adjusted ferritin (below vs above <12 µg/L) | 0.058 |
| Baseline LAZ (below vs above <-1) | 0.298 |
| Baseline actions recalled score (below vs above median) | **0.038*** |
| Maternal age (below vs above 20y) | 0.511 |
| Maternal education (incomplete primary vs primary or greater) | 0.177 |
| FCI score (below vs above median) | 0.503 |
| HOME score (below vs above median) | 0.400 |
| Household food insecurity score (none/mild vs moderate/severe) | 0.289 |
| Household asset index quintile (quintile 1 vs 2-5) | 0.819 |
| *Predictive* |  |
| Group (intervention vs control) | 0.059 |
| Child sex (male vs female) | 0.193 |
| Birth order (first vs not first) | 0.462 |
| Baseline DHA (below vs above median) | 0.570 |
| Baseline adjusted ferritin (below vs above <12 µg/L) | 0.736 |
| Baseline LAZ (below vs above <-1) | 0.217 |
| Baseline actions recalled score (below vs above median) | 0.984 |
| Maternal age (below vs above 20y) | 0.491 |
| Maternal education (incomplete primary vs primary or greater) | 0.190 |
| FCI score (below vs above median) | **0.032*** |
| HOME score (below vs above median) | 0.051 |
| Household food insecurity score (none/mild vs moderate/severe) | 0.734 |
| Household asset index quintile (quintile 1 vs 2-5) | **0.031*** |

^HCAZ – head circumference-for-age z-score; IOWA – Infant Orienting with Attention task; LAZ – length-for-age z-score; MDAT – Malawi Developmental Assessment Tool; VPC – visual paired comparison task; WAZ – weight-for-age z-score^

^a^ The following characteristics were assessed as potential effect modifiers by inclusion of an interaction term: child sex (male vs female), baseline LAZ (≤-1 vs >-1) or stunting status (stunted vs not stunted), baseline maternal age (≤20y vs >20y), baseline housing and asset index (highest quintile vs other quintiles), and time point of data collection (enrollment vs 6 month follow up, for models with both time points). Additionally, longitudinal prevalence of diarrhea (above vs below median) was tested as an effect modifier for growth outcomes. Because the primary developmental analysis suggested an effect of the egg intervention on the most advantaged groups (Prado et al., 2020), additional effect modifiers for developmental outcomes included: group assignment (intervention vs control), birth order (first born vs not first born), maternal education (incomplete primary school vs primary or greater), baseline household food insecurity (mild/no insecurity vs moderate/severe insecurity), baseline developmental score (above vs below median; for elicited imitation, used total MDAT score at enrollment), HOME score (above vs below median), and FCI score (above vs below median). Adjusted plasma ferritin (<12 µg/L vs >12 µg/L (Lynch et al., 2018)) and plasma DHA (above vs below median) were also examined as potential effect modifiers of development due to their connection to choline metabolism and brain development (Kennedy et al., 2014; Mun, Legette, Ikonte, & Mitmesser, 2019). To limit the number of tests, these effect modifiers were only included in minimally adjusted models. Stratified results are only presented for analyses in which the p-for-interaction was statistically significant (p<0.05).
^b^ Continuous growth/developmental outcomes and dichotomous growth outcomes were assessed in generalized linear models and logistic regression models, respectively, with both time points, participant as independent unit, and robust standard errors.

^c^ Conditional growth measures and developmental outcomes were assessed in linear regression models, with baseline plasma metabolite as a predictor.

*p<0.05

References for Supplemental Material

Kennedy, B. C., Dimova, J. G., Siddappa, A. J. M., Tran, P. V., Gewirtz, J. C., & Georgieff, M. K. (2014). Prenatal choline supplementation ameliorates the long-term neurobehavioral effects of fetal-neonatal iron deficiency in rats. *Journal of Nutrition, 144*, 1858-1865. doi:10.3945/jn.114.198739.iron

Lynch, S., Pfeiffer, C. M., Georgieff, M. K., Brittenham, G., Fairweather-Tait, S., Hurrell, R. F., . . . Raiten, D. J. (2018). Biomarkers of Nutrition for Development (BOND)-Iron review. *Journal of Nutrition, 148*, 1001S-1067S. doi:10.1093/jn/nxx036

Mun, J. G., Legette, L. L., Ikonte, C. J., & Mitmesser, S. H. (2019). Choline and DHA in maternal and infant nutrition: Synergistic implications in brain and eye health. *Nutrients, 11*. doi:10.3390/nu11051125

Prado, E. L., Maleta, K. M., Caswell, B. L., George, M., Oakes, L. M., DeBolt, M. C., . . . Stewart, C. P. (2020). Early child development outcomes of a randomized trial providing one egg per day to children age 6 to 15 months in Malawi. *Journal of Nutrition, 150*, 1933-1942.
